# [Retracted] Downregulation of LINC00707 promotes osteogenic differentiation of human bone marrow-derived mesenchymal stem cells by regulating DKK1 via targeting miR-103a-3p

**DOI:** 10.3892/ijmm.2026.5965

**Published:** 2026-08-24

**Authors:** Jun Liu, Minfei Wu, Guang Feng, Rui Li, Yang Wang, Jianhang Jiao

Int J Mol Med 46: 1029-1038, 2020; DOI: 10.3892/ijmm.2020.4672

Following the publication of the above paper, the authors contacted the Editorial Office to explain that they had incorrectly assembled certain of the data included in the western blots featured in Fig. 2D on p. 1033, and wished to issue a corrigendum. Upon performing an independent analysis of the data in this paper in the Editorial Office, however, it came to light that the data in question were strikingly similar to data which had already been submitted to the same journal (*International Journal of Molecular Medicine*) in a paper written by different authors at a different research institute.

Upon asking the authors for an explanation concerning these data, the authors presented to the office a series of blots related to the same experiments, including the purported integral blots associated with the published data. However, owing to a number of remaining uncertainties concerning the blots provided resulting from an additional assessment of the raw underlying data made using the software analysis program, the Editor of *International Journal of Molecular Medicine* has decided that this paper should be retracted from the Journal on account of a lack of confidence in the originally presented data. The Editor apologizes to the readership for any inconvenience caused.

